# Analysis of the Impact Dynamics of Shape Memory Alloy Hybrid Composites for Advanced Applications

**DOI:** 10.3390/ma12010153

**Published:** 2019-01-05

**Authors:** Michele Guida, Andrea Sellitto, Francesco Marulo, Aniello Riccio

**Affiliations:** 1 Department of Aerospace Engineering, University of Naples “Federico II”, 80125 Napoli, Italy; francesco.marulo@unina.it; 2 Department of Engineering, University of Campania “Luigi Vanvitelli”, 81031 Aversa, Italy; andrea.sellitto@unicampania.it (A.S.); aniello.riccio@unicampania.it (A.R.)

**Keywords:** smart materials, shape memory alloy materials, low velocity impact, thermoplastic material

## Abstract

In this work, the behaviour of thermoplastic composites and Shape Memory Alloy Hybrid Composites (SMAHCs) for aeronautical applications is analysed and compared by means of findings from numerical analyses and experimental tests. At first, experimental tests are performed by using a drop tower facility on both carbon fibre reinforced plastic samples and Carbon Fibre Reinforced Plastic (CFRP) samples hybridized with shape memory alloy materials. The materials properties and the different lower velocity impacts behaviours are simulated and validated by means of numerical models discretized in LS-Dyna explicit solver. For both configurations, the deformation mechanism for low intensity impacts, the absorbed energy, and the effect of rebounding upon the velocity change, and hence the amount of force, are investigated. Then, a configuration is prepared to withstand higher-energy impacts. Finally, the numerical analysis is extended for an innovative layup adapted on an aeronautical structure, which is subjected to the bird-strike phenomenon at 180 m/s and with an impacting mass of 1.8 kg according to the airworthiness requirements. In this study, SMAHCs are used to improve the composite impact response and energy absorption thanks to the superelastic effect.

## 1. Introduction

The properties of smart materials can be significantly modified by external causes, such as stress, temperature, humidity, electric or magnetic fields, and for this reason they offer unexpected potential compared to traditional ones [1]. They adapt to the boundary conditions by changing their properties, shape or size simply by adding heat, or moving from a liquid to a solid almost instantly when near a magnet. Among the smart materials, shape memory alloys (SMAs) are characterized by large deformations induced and recovered through changes in temperature or stress [2]. Nitinol was one of the first developed shape memory alloys, originally discovered in 1962 by W.J. Buehler at U.S. Naval Ordinance Laboratory, merging 55% of Nickel and 45% of Titanium material, and named after the composition and the place of development (Nickel Titanium Naval Ordinance Laboratory) [3]. The shape memory alloys have the characteristic to “memorize” and return to their initial configuration when subjected to a temperature higher than their characteristic transition temperatures. Indeed, the SMA microstructure is subjected to a phase transformation, from martensite to austenite, induced by the transition temperature. Furthermore, the possibility of being coupled with other materials that have a different peculiarity allows one to tailor the properties according to the desired material application [4].

This paper summarizes research on improving the impact performance of polymer matrix composites by embedding shape memory alloys, in order to increase the level of absorbed energy before failure, mainly ascribed to the ability of SMA materials to decrease the damage area resulting from an impact event, but also due to the ability to undergo large amounts of elastic and plastic deformation, at moderately high stresses, due to the superelastic properties of TiNi shape memory alloy.

Birman et al. [5] used a micromechanical model based on the multi-cell method to model pre-strained SMA enhanced FRPs, while Tsoi et al. [6], demonstrated by experimental tests that embedding different types of wires in a composite structure improves the resistance, in particular, to fibre breakage damage.

The composite materials are not subjected to relevant indentation damages in case of low-velocity/energy impacts. Indeed, the contact and global deformations are comparable, with the contact deformation being the difference between the impactor and the test sample’s back-face displacements [7]. The Hertz contact law defines the relationship between the contact deformation and the contact force, and the modified Hertz law, introduced by Sun [8], formulates a contact between a composite laminate and a rigid sphere or cylinder. Since no exact elasticity solutions is available for the contact of general composite laminates, Hertz law and modified Hertz laws are used for modelling purposes. The Hertz law overestimates the impact force, since it considers only the contact deformation for plates supported at external boundaries. On the other side, the modified Hertz law additionally includes the flexural displacement, giving a more accurate prediction of the impact force.

Polymer-based composites are characterized by high strength and stiffness associated with a low weight [9,10,11]. However, they have relatively limited applications due to their brittle behaviour, related to matrix–fibre interaction, with consequent poor performances in terms of damage resistance to impact if compared to ductile materials. Indeed, unlike metallic materials absorbing the impact energy through plastic deformation, no plastic deformation can be observed in composites during impact. However, composite materials have been found able to offer the advantage of an increasing absorption for impact energy by properly combining several materials. Indeed, additional components can be added to the composite, increasing the impact resistance without compromising the desired mechanical properties of the resulting “hybrid” composite, such as graphene nanolayers, single and multi-walled carbon nanotubes, hollow fibres, and through-the-thickness reinforcement [12,13]. Among the possible solutions, composites can be hybridised by embedding shape memory alloys characterised by a superelastic behaviour, resulting in an increase of the impact damage resistance [14,15].

In order to fully take advantage of SMAs’ potential in engineering applications, a comprehensive understanding of the SMA mechanical behaviour is needed. In [16], an extensive test campaign was performed to assess the stiffness and strength properties of the reinforced carbon fibre (CF) thermoplastic and of the SMAHC hybrid material based on Polyphenylene Sulphide (PPS) matrix. The impact test results, performed at different energy levels, showed that hybrid specimens have a greater ability to absorb impact energy compared to traditional thermoplastic composites structures and to reduce the risk of delamination arising from the impact.

In this paper, an experimental-numerical investigation on the impact damage behaviour of CFRP (Carbon Fibre Reinforced Plastic) composite plates with embedded SMAs is presented. The results demonstrated that hybrid composites structures embedded with shape memory alloys subjected to low velocity impacts are characterized by an increased damage resistance with respect to conventional composite structures. Indeed, kinetic energy is absorbed by the SMA wires during the impact, thanks to their superelastic and hysteretic behaviour. In particular, the superelastic effect is related to reversible stress-induced transformation from austenite to martensite. Applying a stress to the alloy during its austhenitic state will results in large deformation strains and to the formation of stress induced martensite. Moreover, once the stress is removed, the martensite returns to its austenitic parent phase. Therefore, the SMA undergoes a large hysteresis loop, obtaining a large recoverable strain. Thus, the impact energy is evenly distributed in the specimens by the hybrid fabric, as confirmed by the presented experimental tests.

## 2. Hybrid Materials Properties

**Figure 1:**
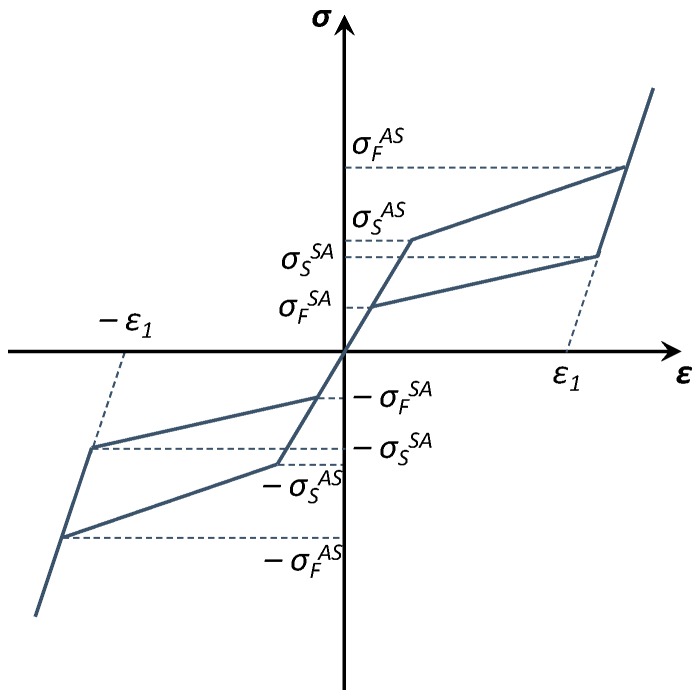
Theoretical stress-strain curve for a shape memory alloy (SMA).

The typical stress-strain relationship of a SMA can be appreciated in Figure 1. Indeed, the material properties at working temperatures are strongly influenced by the SMA phase transformation. This effect cannot be considered in the stress-strain constitutive models of conventional materials. Hence, as already mentioned, a good understanding of the mechanical behaviour and an accurate constitutive modelling of the SMA are mandatory for the development of further applications. Different approaches can be found in literature to describe the SMA constitutive models, such as macroscopic, microscopic, and mixed. According to the macroscopic approaches, the material behaviour is described by the constitutive model on the basis of experimental data. On the other hand, microscopic approaches, the material behaviour is described by the constitutive model based on concepts derived from the fundamental physical, such as the microstructure of the crystal lattice. Finally, in the mixed approaches, both macroscopic and microscopic approaches are used for improving modelling.

Based on their complex stress-strain relationship, SMA wires can be embedded in composite structures to improve the impact damage resistance. A superelastic shape memory alloy is characterized by high strain capability, which is mainly related to the plateau region in the stress-strain relation due to the stress-induced martensitic phase transformation. Indeed, high strains to failure and recoverable elastic strains (up to 15%) can be observed in the SMA fibres. Therefore, SMA fibres are able to absorb higher strain energy before failure compared to other fibres. For such a reason, composites with embedded superelastic SMA fibres are characterized by an increase impact damage resistance. A review on the mechanics, the properties, and the applications of shape memory alloys can be found in [17]. In [6], the effects of embedded SMA wires in composites subjected to low velocity impacts were assessed. The results demonstrated that SMA wires did not compromise the structure, improving, in some cases, the damage resistance. In [18], SMA wires were stitched into the composite to reduce the possibility of impact-induced delaminations. In particular, experimental and theoretical studies on SMA stitched glass/epoxy composites, subjected to low velocity impact, showed that stitching the composite plates by SMA wires resulted in an increase of the strength and a reduction of the number of translaminar cracks. Moreover, according to the theoretical studies, the superelastic SMA wires absorbed energy, resulting in a reduction of the delamination energy of the stitched composite plates. Another study on the increase of the damage behaviour of laminates subjected to impact phenomena by means of embedded superelastic SMA can be found in [19]. According to the study, SMA wires embedded in the laminate can improve the impact resistance, preventing a complete perforation of the laminate.

In this paper, a numerical investigation on the impact damage resistance of hybrid composites plates with embedded SMA wires is presented. The SMA wires are embedded into a multiangle ply lay-up and the optimal location of SMA wires to be used is assessed.

Three different regions can be identified in the typical SMA stress-strain curve in tension and compression, shown in Figure 1, where:

*σ_S_^AS^* = start of the martensitic state;
*σ_F_^AS^* = finish of the martensitic state;
*σ_S_^SA^* = start of the austenitic state;
*σ_F_^SA^* = finish of the austenitic state.

Indeed, a pure elastic behaviour can be observed below *σ_S_^AS^*. Higher values of stress will lead to the initiation of the forward transformation with the formation of stress-induced martensite, generating large-transformation strains as in the upper plateau of the stress-strain curve. The forward transformation is completed once the value *σ_F_^AS^* of the applied stress is reached. The SMA is in its martensitic phase. A pure elastic martensitic behaviour is observed applying a load above *σ_F_^AS^* value. The reverse transformation of the martensite into austenite initiates upon unloading. The reverse transformation starts at a value *σ_S_^SA^* of the stress and it is completed at a value *σ_F_^SA^* of the stress. The difference between the stresses *σ_F_^AS^* and *σ_S_^SA^* and the stresses *σ_S_^AS^* and *σ_F_^SA^* results in the hysteretic loop reported in the loading/unloading stress-strain curve [20].

The characteristic of the 0.5 mm diameter Nitinol alloy wires used for the applications presented in this paper are reported in Table 1 [21].

## 3. Experimental Tests

Low velocity impact tests were performed on two different configurations: thermoplastic PPS/CF square specimens with a theoretical thickness of 2.17 mm, and thermoplastic PPS/CF square specimens hybridised with 2.53 mm theoretical thickness SMA wires.

The semipregs PPS/CF (Cetex^®^ PPS) are composed by dry fabric and resin films fused on the outside, defined in literature as a CFRP with reinforced thermoplastic matrix in the form of prepreg. The SMA wires were coupled to the semipreg layers and to the hybrid fabric thanks to a 0.127 mm-thickness PPS film, used to help the adhesion. The hybrid fabric was realized by hybridising an aramid warp knitted biaxial tape, produced by crochet knitting, with SMA wires and PPS filaments placed along the fabric longitudinal direction.

To clarify the manufacturing process, good carbon fabric impregnation was obtained thanks to an isothermal compression moulding process, which minimized the void percentage; however, the process needs long cycle-times, considerably increasing the operational costs. Both heating and cooling phases occur under the press, without a material preliminary heating phase, as described in ref. [14].

**Figure 2:**
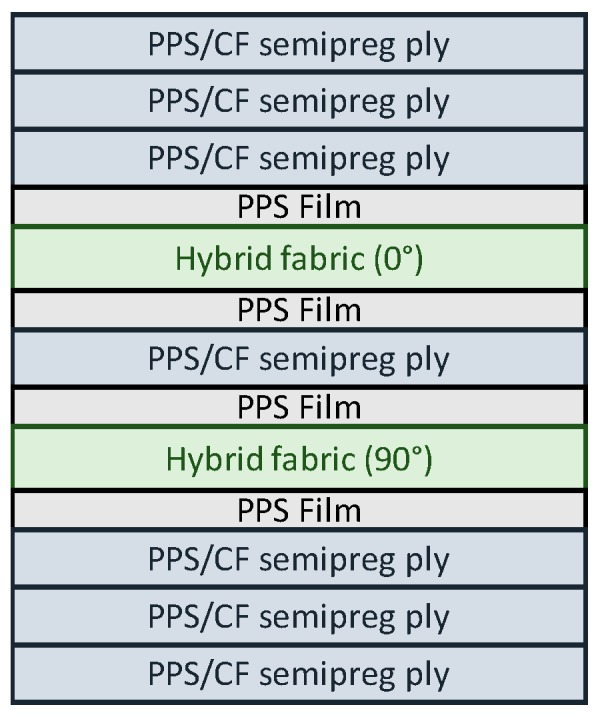
Lamination sequence of hybrid samples (not in scale).

The thermoplastic laminates are composed of seven (7) semipreg layers (Cetex^®^ PPS). Finally, the hybrid SMA laminates lamination sequence is reported in Figure 2.

The thermoplastic semipregs and the SMA hybrid fabric were cut and stacked following the lamination sequence reported in Figure 2; the layers were inserted into the press and the plates were closed. The guidelines that were followed for performing the consolidation process are reported below:

1. step 1: heat the material up to 305–310 °C at low pressure (about 2 bar), the heating rate should be very high (the maximum achieved by the equipment);
2. step 2: keep the material at 305–310 °C for 75 minutes at low pressure (about 2 bar);
3. step 3: keep the material at 305–310 °C for 15 minutes at high pressure (about 30 bar) with regular degasification steps;
4. step 4: cool the material up to the environment temperature with a rate of 3 °C/min (keeping the pressure at 30 bar).

When the moulding process was completed, laminates were cut obtaining a size of 200 × 200 mm^2^.

The obtained samples were characterized by very good impregnated SMA wires, thanks to the adopted manufacturing process, as demonstrated by the coupons’ thicknesses, which are close to the theoretical value.

Low velocity impact tests were conducted using a drop tower. Moreover, a load cell was used to acquire the Force-time history. Considering a falling mass of 6.84 kg, the values for impact energy and impact velocity are summarized in Table 2.

The specimens were clamped during the impact test to reduce movements and vibrations. It is worth noting that the final results are strongly affected by the type of clamp. For this reason, different clamping conditions may lead to different test results.

**Figure 3:**
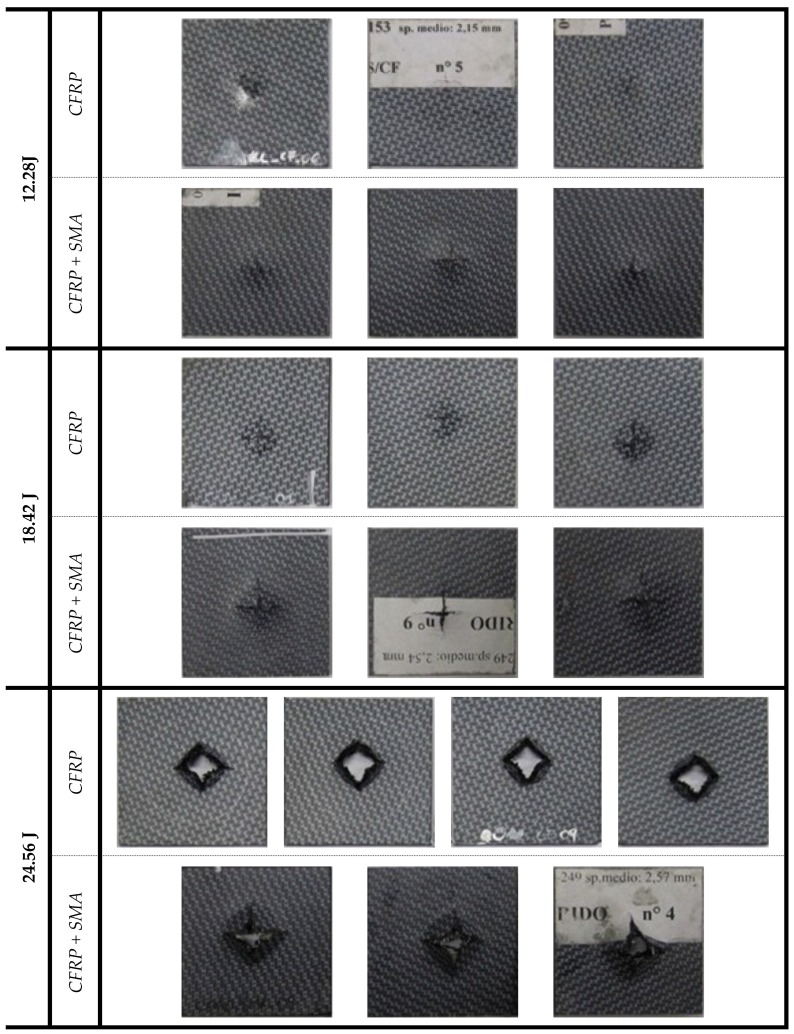
Inspection of the impacted samples.

In order to evaluate the different ways to reacts to the impact dynamics, a first correlation has been considered to evaluate the damage development in the CFRP unreinforced samples and in the CFRP+SMA hybrid samples subjected to impacts shown in Figure 3. The distribution of the damages induced by impact phenomena is generally very complex, due to the different failure modes interacting with each other. Moreover, the impact damage evolution cannot be predicted using only failure criteria or fracture mechanics approach. The onset of the damage can be predicted by using failure criteria based on the impact force and stress. Despite its complexity, the problem can be still analysed by means of composite plate or beam theory, fracture mechanics, failure theory of composites, contact mechanics, etc., since the composite structures still maintain their intact shape [22].

The residual properties of the structure strongly depend on the final damage state. However, it is not possible to predict the damage in detail, since the strength of the structure, particularly the ones subjected to compressive loads, is mainly reduced by the delaminations. Therefore, an accurate prediction of the delamination size is mandatory.

## 4. Experimental Results

**Figure 4:**
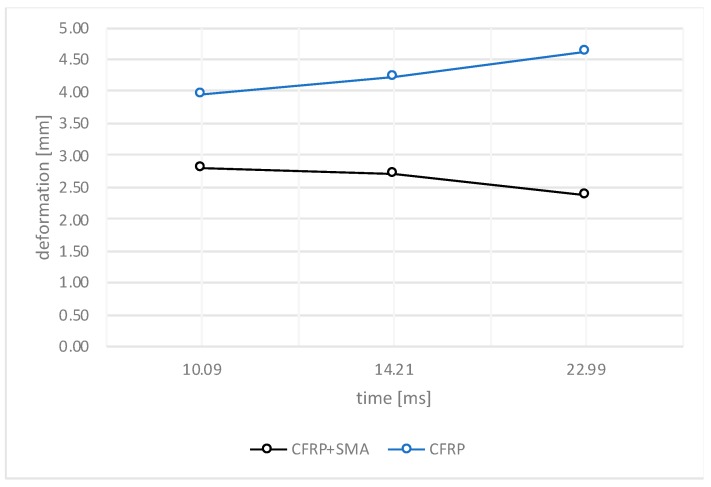
Deformation of the impacted samples.

**Figure 5:**
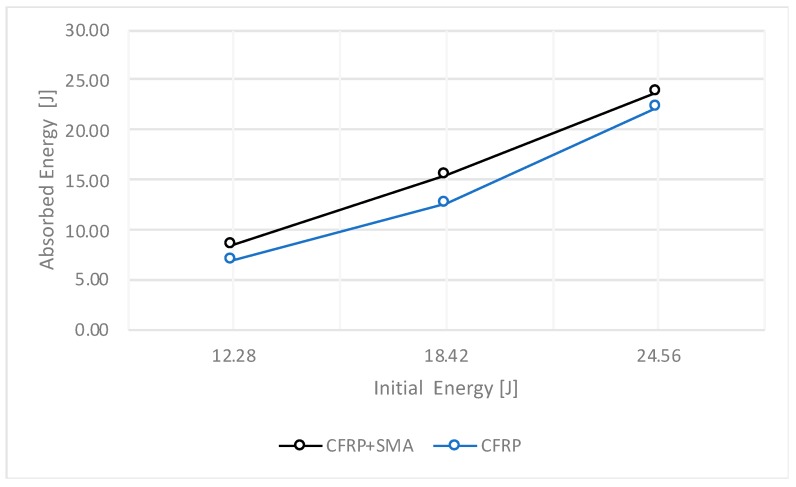
Energy history.

**Figure 6:**
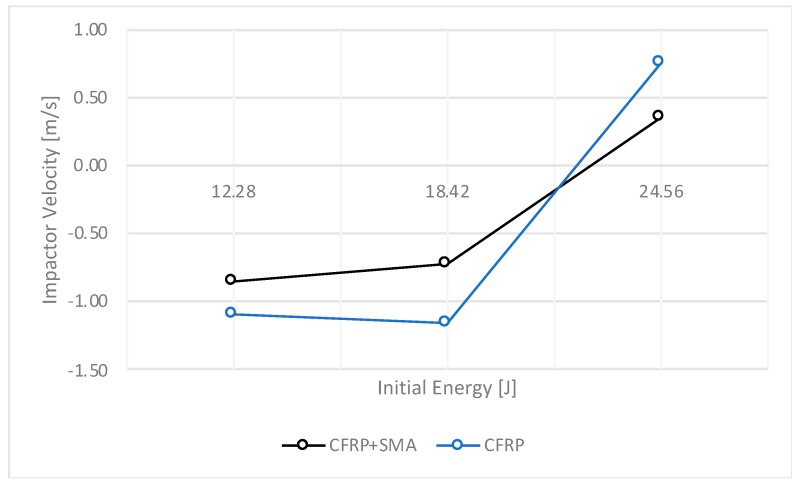
Impactor rebound velocity.

**Figure 7:**
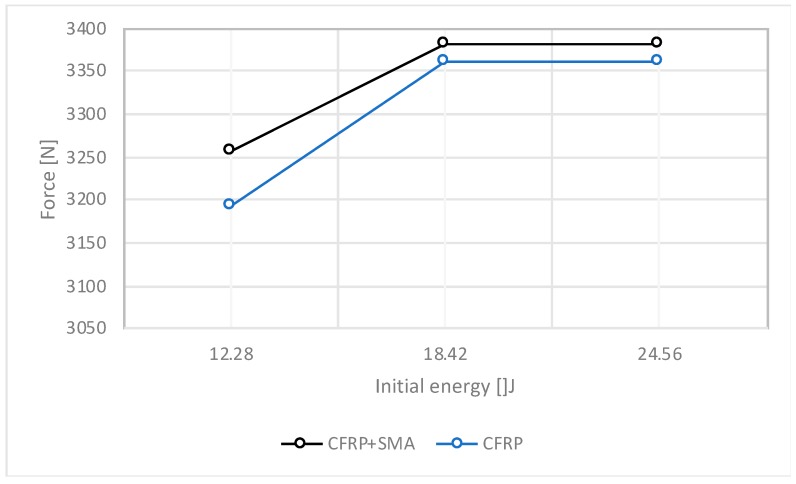
Comparison of the maximum force between PPS/carbon fibre (CF) and hybrid composite.

In Figure 4, Figure 5, Figure 6 and Figure 7, the data collected for the CFRP samples and for the CFRP+SMA hybrid composites resulting from the impact tests are shown. In particular, each point reported in Figure 4, Figure 5, Figure 6 and Figure 7 corresponds to a single experimental measurement.

The experimental tests were performed using a CEAST instrumented drop tower machine conforming to certified technical standards. Figure 4 shows the results, in terms of maximum attained displacement when the peak force is reached on the samples, for the CFRP baseline and hybrid specimens considering three different impact energies (12.28 J, 18.42 J, and 24.56 J) and measured by striker during the impact. According to the results, the baseline samples are characterized by higher values of the displacements as the impact energy increases. On the contrary, the hybrid materials present a reduced displacement for the higher levels of the impact energy, probably due to the presence of the SMA wires that reinforce the layup and, as observed in the Figure 3, because a wider portion of the panel absorbs the impact energy.

Figure 5 reports the behaviour of the energy transferred from the impactor to the sample for both CFRP and hybrid samples. As it is possible to observe, an increase of the incidental energy results in an almost linear increase of the quantity of energy absorbed during the impact event for both the tested composites. Moreover, the global absorbed energy from the hybrid samples is higher than the baseline sample. Additionally, for the maximum value of initial energy (24.56 J), the hybrid sample absorbed 97% of the initial energy, compared to the 90% of the energy absorbed by the baseline sample.

Important considerations can be drawn considering the trend of the impactor velocity as a function of the impact energy shown in Figure 6, which reports the velocity recorded from the impactor after the impact for both the baseline and hybrid composites. It is possible to notice that for first two levels of the initial energy (12.28 J and 18.42 J), the impactor rebounds with a reduced velocity which depends on the penetration depth. Moreover, in the case of the highest energy level considered (24.56 J), an increase of the number of fibres actually broken by the head of the impactor is observed. Indeed, the specimens are perforated and no partial rebound is observed, assuming the shape of a “volcano”, as shown in Figure 3.

The comparison between the maximum attained force as a function of the impact energy for both the hybrid composite and the CFRP baseline is shown in Figure 7. Indeed, for each impact energy, the SMA-based hybrid composites are characterized by higher maximum forces compared to the baseline ones. This result suggests that the stiffness of the composite is improved by the insertion of the SMA within the laminate, resulting in a material able to withstand higher-energy impacts.

The extensive experimental testing carried out at low velocity impacts has been replicated by the numerical simulations, where the behaviour of the hybrid samples was reproduced by the material model that describes the superelastic response present in shape-memory alloys, that is the peculiar material ability to undergo large deformations with a full recovery in loading-unloading cycles. The validations with the case study tuned the SMAHC material in order to evaluate and apply them in advanced applications.

## 5. Advanced Application for the SMA Hybridized Composite Materials

**Figure 8:**
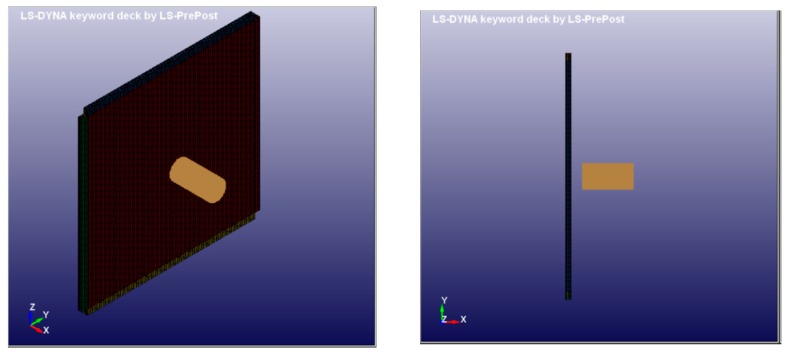
Simplified model to estimate the bird-strike.

Following several studies in the field of smart materials and structure, a possible application is to design an aeronautical structural component (as a leading edge) subjected to the bird strike to evaluate the impact resistance performances [23,24]. In this section, the numerical results of the bird strike on the simplified square plate are presented. The square plate is modelled by adopting the LS-DYNA Explicit FEM code, as shown in Figure 8.

**Figure 9:**
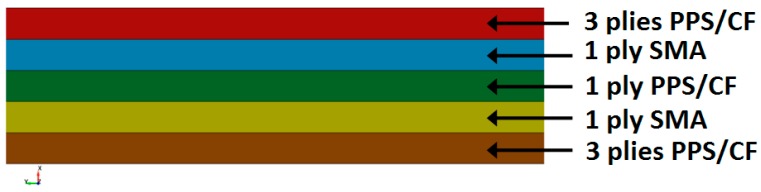
Layup configuration of the simplified model.

A cylinder with length-to-diameter ratio of two has been used to model the 1.8 kg (4 lb) bird. A diameter of 0.098 m has been considered. The incidental velocity of the soft projectile is equal to 180 m/s. The 1 m^2^ flat target is impacted with a 90° angle respect to the horizontal x-axis. In Figure 9, the layup configuration of the coupon used in the material characterization is shown. The thickness of each PPS/CF ply is equal to 0.31 mm, while the thickness of each SMA ply is equal to 0.2 mm, leading to nominal total thickness of 2.57 mm.

This configuration presents the best layup, chosen as a compromise in terms of stiffness, weight, and ability to withstand to a highly transitory phenomenon like the bird-strike scenario, where a total perforation is not contemplated, and the deformations must be contained in order not to compromise the integrity of what is behind the panel.

**Figure 10:**
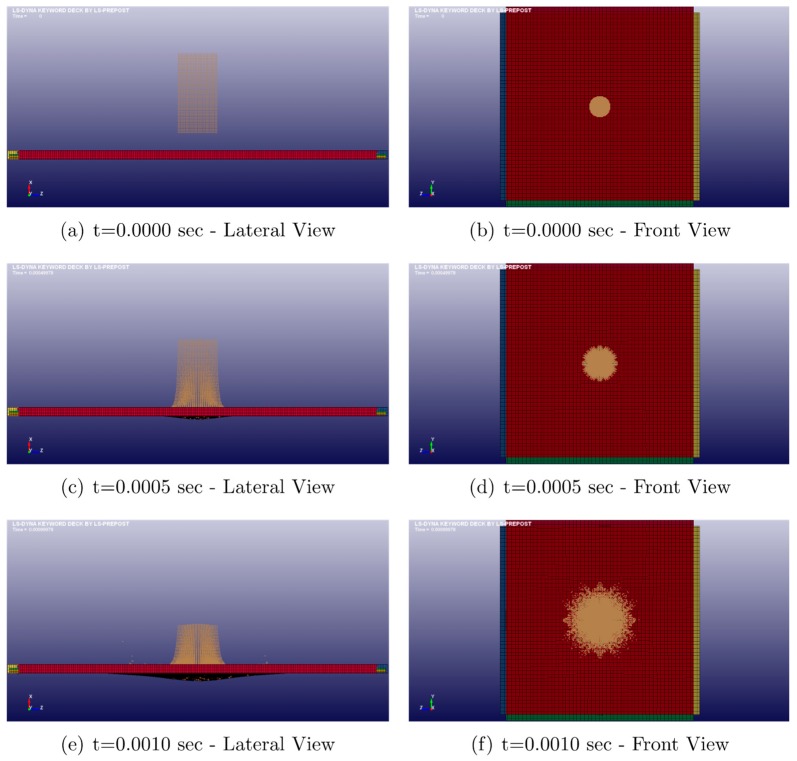

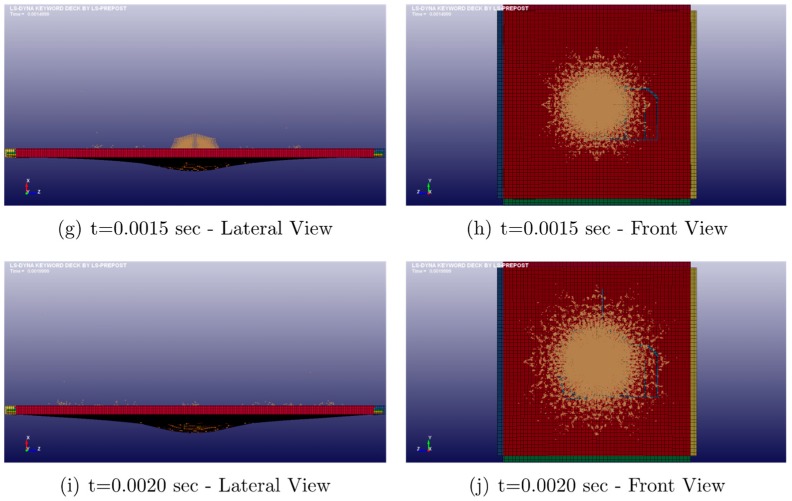
Sequence of plate deformation up to 2 ms

Figure 10 shows the deformation of the impacted panel, from the beginning of the impact up to 2 ms. The panel experiences complete perforation, while the bird is completely squashed.

**Figure 11:**
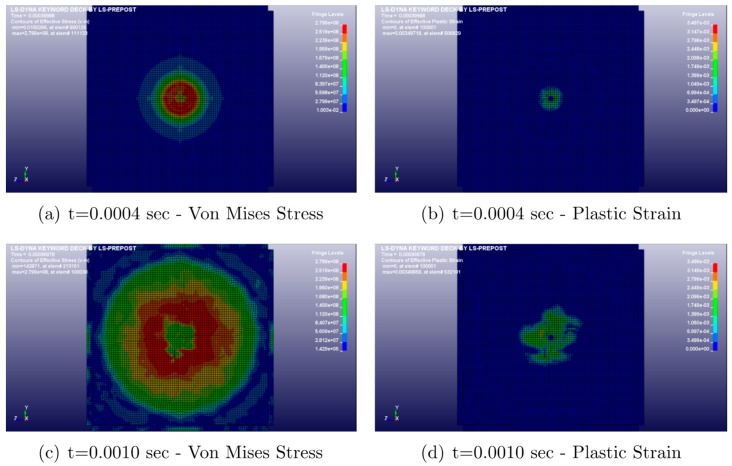

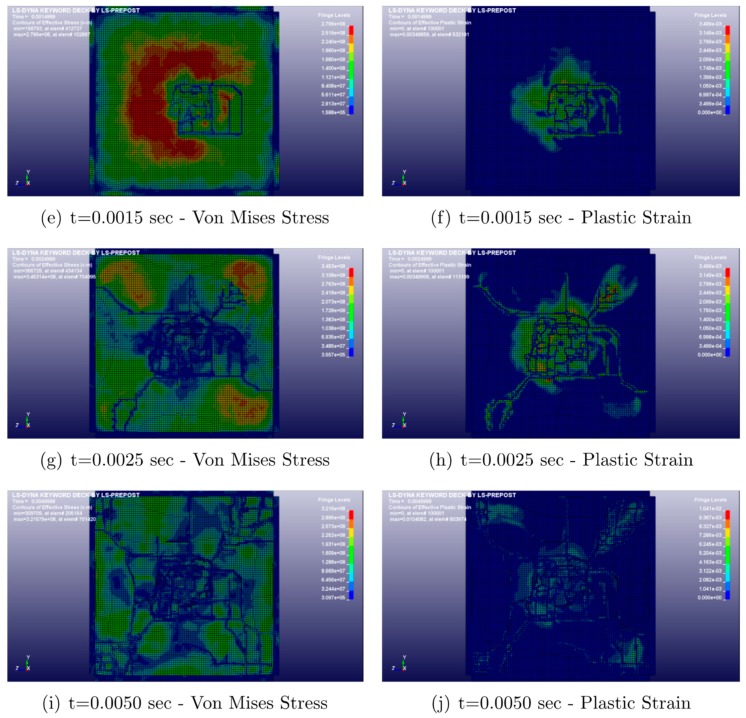
Von Mises stress and plastic strain.

Almost 90% of the bird’s load is transferred to the plate during the initial 2 ms, with a consistent deformation of the centre of the plate, corresponding to the bird-impacted region. Just before the perforation, no expansion of the plate deformation from the central region to the plate edges can be observed. This occurs despite the presence of SMA interlayer, which does not contribute to a more extensive area of the panel to absorb the impact energy. Figure 11 reports the contour plots of the von Mises stress and of the plastic strain for different times.

In this case, during the beginning of the impact, only the central region of the plate is involved in the phenomenon, reaching a 280 MPa peak value of the von Mises stress. Additionally, after 1.5 μs, an evident failure of the plate can be observed, which propagates to the bottom right corner. Then, only the intact zone of the plate maintains load-carrying capability.

**Figure 12:**
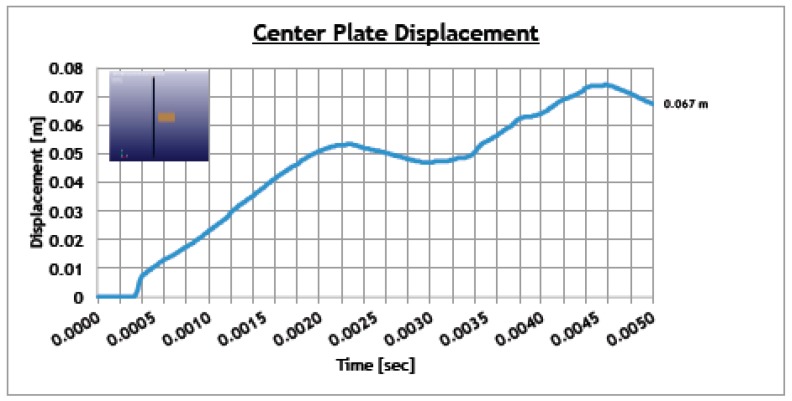
Central node displacement.

Figure 12 reports the resultant displacement for the central plate element (which experiences the impact). At the end of the simulation, a displacement of 67 mm is obtained.

In the case of the aircraft leading edge, a skin’s large deformation can compromise the integrity of the front spar that represents the primary structure installed behind the skin, so this value is considered a good compromise in absence of the total perforation.

In bird-strike analyses, the initial energy is only represented by kinetic energy of the bird which is equal to:

(1) $$E_{kin}=\frac{1}{2}mv^{2}=21.6\;\mathrm{kJ}$$

**Figure 13:**
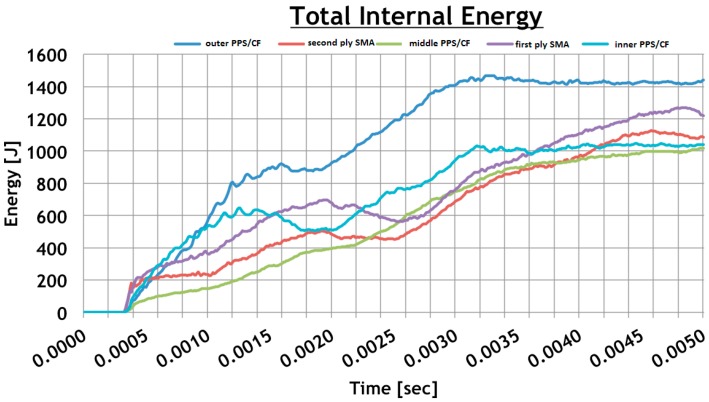
Total internal energy for each layer.

Figure 13 reports the time-histories of the internal energy for each layer.

According to Figure 13, this configuration is able to absorb 25% of the energy while the residual part is associated with the squashing of the bird onto the panel. Furthermore, the outer PPS/CF layer, the one directly impacted by the bird, absorbs the higher amount of the bird’s impact energy. However, the two SMA layers are found to be very efficient at absorbing energy, thanks to their elastic behaviour.

## 6. Conclusions

This paper reports an experimental-numerical study aimed at the mechanical characterization of a composite laminate composed of thermoplastic resin reinforced with carbon layers and layers consisting of a fabric hybridized with shape memory alloy. Experimental impact tests were carried out on both CFRP and hybrid specimens in order to assess the influence of the hybrid fabric on the impact behaviour of structures. The low velocity impact tests have demonstrated the greater energy absorbing capability during the impact of the specimens containing the hybrid layers. These experimental tests indicated that both the material systems absorb the same amount of energy when subjected to low velocity impacts (12.28 J and 18.42 J). However, the delaminated area of the CFRP specimens is much more extended respect to the hybrid ones. Therefore, the hybridization process changes the energy absorption mechanisms of the structure modifying the response to low velocity impacts for the reinforced material. The behaviour of the hybrid composites can be related to the superelastic and hysteretic properties of the SMA wires included within the structure. Indeed, the energy dissipated through intra-laminar damages is reduced by the large amount of energy absorbed by the SMA during the impact.

Finally, this material configuration was applied to the design of a structural aeronautical component to satisfy the bird-strike requirement. According to the numerical results, the structure has demonstrated its suitability for this purpose.

## Figures and Tables

**Table 1:** Mechanical properties of the shape memory alloy adopted for the wires.

Density [g/cm^3^]	Young Modulus [GPa]	Poisson’s Ratio [-]	*σ_F_^AS^* [MPa]	*σ_S_^AS^* [MPa]	*σ_S_^SA^* [MPa]	*σ_F_^SA^* [MPa]	*ε*_1_ [mm/mm]
6.45	29.3	0.33	896	790	600	450	0.078

**Table 2:** Specification for low velocity impact tests.

Impact Energy [J]	Velocity [m/s]
12.28	1.89
18.42	2.32
24.56	2.68

## References

[1] Brostow W., Lobland H.E.H. (2017). Materials: Introduction and Applications.

[2] Yang H., Fortier A., Horne K., Brostow W., Lobland H.E.H. (2016). Shape memory metal alloys in the context of teaching smart materials. J. Mater. Educ..

[3] Buehler W.J., Wiley R.C. (1965). Nickel-Based Alloys. U.S. Patent.

[4] Angioni S.L., Meo M., Foreman A. (2011). Impact damage resistance and damage suppression properties of shape memory alloys in hybrid composites—A review. Smart Mater. Struct..

[5] Birman V. (1997). Review of mechanics of shape memory alloy structures. Appl. Mech. Rev..

[6] Tsoi K.A., Stalmans R., Schrooten J., Wevers M., Mai Y.-W. (2003). Impact damage behaviour of shape memory alloy composites. Mater. Sci. Eng. A.

[7] Tracy J.J., Dimas D.J., Pardoen G.C. The effect of impact damage on the dynamic properties of laminated composite plates. Proceedings of the 5th International Conference on Composite Materials, ICCM-V.

[8] Sun C.T., Chattopadhyay S. (1975). Dynamic response of anisotropic plates under initial stress due to impact of a mass. J. Appl. Mech..

[9] Sellitto A., Riccio A., Russo A., Zarrelli M., Toscano C., Lopresto V. (2019). Compressive behaviour of a damaged omega stiffened panel: Damage detection and numerical analysis. Compos. Struct..

[10] Riccio A., Cristiano R., Saputo S., Sellitto A. (2018). Numerical methodologies for simulating bird-strike on composite wings. Compos. Struct..

[11] Riccio A., Raimondo A., Saputo S., Sellitto A., Battaglia M., Petrone G. (2018). A numerical study on the impact behaviour of natural fibres made honeycomb cores. Compos. Struct..

[12] Riccio A., Linde P., Raimondo A., Buompane A., Sellitto A. (2017). On the use of selective stitching in stiffened composite panels to prevent skin-stringer debonding. Compos. Part B Eng..

[13] Tessitore N., Riccio A. (2006). A novel FEM model for biaxial non-crimp fabric composite materials under tension. Comput. Struct..

[14] Rogers C.A., Robertshaw H.H. (1988). Shape Memory Alloy Reinforced Composites. Eng. Sci. Prepr..

[15] Guida M., Marulo F., Russo S. (2018). NiTi SMA wires coupled with Kevlar fabric: A real application of an innovative aircraft LE slat system in SMAHC material. Appl. Compos. Mater..

[16] Meo M., Marulo F., Guida M., Russo S. (2013). Shape memory alloy hybrid composites for improved impact properties for aeronautical applications. Compos. Struct..

[17] Birman V., Chandrashekhara K., Sain S. (1996). An approach to optimization of shape memory alloy hybrid composite plates subjected to low-velocity impact. Compos. Part B Eng..

[18] Lau K.-T., Ling H.-Y., Zhou L.-M. (2004). Low velocity impact on shape memory alloy stitched composite plates. Smart Mater. Struct..

[19] Paine J.S.N., Rogers C.A. (1994). The Response of SMA Hybrid Composite Materials to Low Velocity Impact. J. Intell. Mater. Syst. Struct..

[20] Meo M., Antonucci E., Duclaux P., Giordano M. (2005). Finite element simulation of low velocity impact on shape memory alloy composite plates. Compos. Struct..

[21] Khalili S.M.R., Shokuhfar A., Malekzadeh K., Ghasemi F.A. (2007). Low-velocity impact response of active thin-walled hybrid composite structures embedded with SMA wires. Thin-Walled Struct..

[22] Wang C.Y., Yew C.H. (1990). Impact damage in composite laminates. Comput. Struct..

[23] Abrate S. (2005). Impact on Composite Structures.

[24] Aymerich F., Meili S. (2000). Ultrasonic evaluation of matrix damage in impacted composite laminates. Compos. Part B Eng..

